# CT and MRI Determination of Intermuscular Space within Lumbar Paraspinal Muscles at Different Intervertebral Disc Levels

**DOI:** 10.1371/journal.pone.0140315

**Published:** 2015-10-12

**Authors:** Xuefei Deng, Youzhi Zhu, Shidong Wang, Yu Zhang, Hui Han, Dengquan Zheng, Zihai Ding, Kelvin K. L. Wong

**Affiliations:** 1 Anatomical Institute of Minimally Invasive Surgery, Southern Medical University, Guangzhou, Guangdong, China; 2 Department of Anatomy, Anhui Medical University, Hefei, Anhui, China; 3 Department of Radiology, the 105th Hospital of PLA, Hefei, Anhui, China; 4 Department of Orthopaedic Surgery, Shucheng People’s Hospitial, Shucheng, Anhui, China; 5 School of Medicine, Western Sydney University, Campbelltown, New South Wales, Australia; Shenzhen institutes of advanced technology, CHINA

## Abstract

**Background:**

Recognition of the intermuscular spaces within lumbar paraspinal muscles is critically important for using the paramedian muscle-splitting approach to the lumbar spine. As such, it is important to determine the intermuscular spaces within the lumbar paraspinal muscles by utilizing modern medical imaging such as computed tomography (CT) and magnetic resonance imaging (MRI).

**Methods:**

A total of 30 adult cadavers were studied by sectional anatomic dissection, and 60 patients were examined using CT (16 slices, 3-mm thickness, 3-mm intersection gap, *n* = 30) and MRI (3.0T, T2-WI, 5-mm thickness, 1-mm intersection gap, *n* = 30). The distances between the midline and the superficial points of the intermuscular spaces at different intervertebral disc levels were measured.

**Results:**

Based on study of our cadavers, the mean distances from the midline to the intermuscular space between multifidus and longissimus, from intervertebral disc levels L1–L2 to L5–S1, were 0.9, 1.1, 1.7, 3.0, and 3.5 cm, respectively. Compared with the upper levels (L1–L3), the superficial location at the lower level (L4–S1) is more laterally to the midline (*P*<0.05). The intermuscular space between sacrospinalis and quadratus lumborum, and that between longissimus and iliocostalis did not exist at L4–S1. The intermuscular spaces in patients also varied at different levels of the lumbar spine showing a low discontinuous density in CT and a high signal in MRI. There were no significant differences between the observations in cadavers and those made using CT and MRI.

**Conclusion:**

The intermuscular spaces within the paraspinal muscles vary at different intervertebral disc levels. Preoperative CT and MRI can facilitate selection of the muscle-splitting approach to the lumbar spine. This paper demonstrates the efficacy of medical imaging techniques in surgical planning.

## Introduction

Contrasting to open surgery, minimally invasive posterior approaches to the lumbar spine have a major advantage of limiting iatrogenic trauma to surrounding structures, thereby reducing postoperative pain, facilitating patient recovery and shortening hospital stay. Paraspinal approaches allow access to the transverse processes and facets of the lumbar spine, and have been utilized in a variety of surgical procedures used in the management of various spinal pathologies. This includes discectomy for disc herniation, decompression for spinal canal stenosis, insertion of pedicle screws for fractures, lumbar interbody fusions, and treatment of intradural lesions such as tumors [1–11].

Recognition of the intermuscular spaces within the paraspinal muscles is critically important for the paramedian muscle-splitting approach to the lumbar spine. It is generally accepted that the major muscles of the lumbar spine can be divided into the anterior and posterior groups [12]. The anterior group is composed of two major muscles: psoas major and quadratus lumborum. The posterior group comprises the medial multifidus and lateral sacrospinalis; the latter muscle consists of two parts: medial longissimus and lateral iliocostalis [13, 14]. Accordingly, three natural intermuscular spaces exist within their neighboring muscles. We abbreviate them as: ML between the multifidus and longissimus, SQ between the sacrospinalis and quadratus lumborum, and LI between the longissimus and iliocostalis.

These intermuscular spaces provide the natural cleavage planes for the paramedian muscle-splitting approach to the lumbar spine. Three paraspinal approaches through different planes have been described in the literature. In 1959, Watkins stated that SQ was particularly identifiable at the level of L4–L5, and required some resection of the ilium for proper muscle reflection in the procedure [15, 16]. Similarly, Wiltse also concluded that only the L5 and S1 transverse and articular processes can be reached easily through the ML [17, 18]. More recently, Weaver described a modified trans-muscular paraspinal approach via LI, which provided much less invasive access to the lateral spine from L3 to S2 [19].

These reports raise a fundamental question: do these intermuscular spaces exist only in the lower lumbar region and differ in configuration at different intervertebral disc levels? Any differences would indicate that the various levels of the lumbar spine require different operative approaches and skin incisions. Detailed knowledge of the anatomy of the intermuscular spaces can provide guidance for the surgeon and helps to optimize the location and minimize the extent of the required incision for a particular surgical procedure. Therefore, the aim of this study was to determine the intermuscular spaces within the lumbar paraspinal muscles at different intervertebral disc levels with the use of modern medical imaging such as computed tomography (CT) and magnetic resonance imaging (MRI). Numerous other studies have investigated the anatomic properties of these intermuscular spaces with the goal of providing surgical guidelines [20–23], and a recent study demonstrated that ML was well-visualized in patients [24]. As such, the results from our study may facilitate preoperative selection of the appropriate paramedian muscle-splitting approach to the lumbar spine.

## Materials and methods

### Study Subjects

The study was approved by the Ethics Committee of Anhui Medical University (Hefei, China). A total of 30 adult cadavers (17 males, 13 females; age range, 18–74 years; mean age, 49 years) and 60 patients (33 males, 27 females; age range, 18–82 years; mean age, 47 years) were examined in this study.

The cadavers assigned to this project were those used for research and educational purposes by the Department of Anatomy at our institution, and informed written consent was obtained from the relatives of all the deceased. In all cadavers included in this study, the entire vertebral column was present, and there was no evidence of herniation or obvious injuries to the lower back.

All patients included in this study provided informed written consent, and were investigated at the Department of Radiology, Affiliated PLA 105 Hospital of Anhui Medical University between May 2014 and May 2015. The inclusion criteria were: lumbodorsal pain; suspected intervertebral disc herniation; and all lumbar segments completely scanned. The exclusion criteria were: vertebral deformation; any obvious muscular abnormality; previous surgery to the lower back; and a history of trauma.

### Assessment of intermuscular spaces

#### Adult Cadavers

The cadavers were fixed within 36 hours of death by perfusion of 10% formalin solution via the right femoral artery. A cross-sectional study was conducted on the posterior midline region of the lumbar spine to acquire detailed anatomy of the complex architecture of the muscle fascia planes *in situ*. The trunks of the 30 cadavers were kept below –70°C for 6 days in 10% gelatin solution in the supine position, and then anatomically dissected using transverse sections that were 0.3–0.4 cm thick. These dissected specimens were subsequently examined and photographed. Serial slices extending from the L1 to the S1 vertebrae were selected for study of their tissues. Emphasis was placed on the morphology of the natural planes of the paraspinal muscles at different levels and the relationship between the muscles and lumbar fascia. The distances between the midline and the superficial point of the intermuscular spaces at different intervertebral disc levels were measured and recorded.

#### Computed tomography scans of live patients

**Fig 1:**
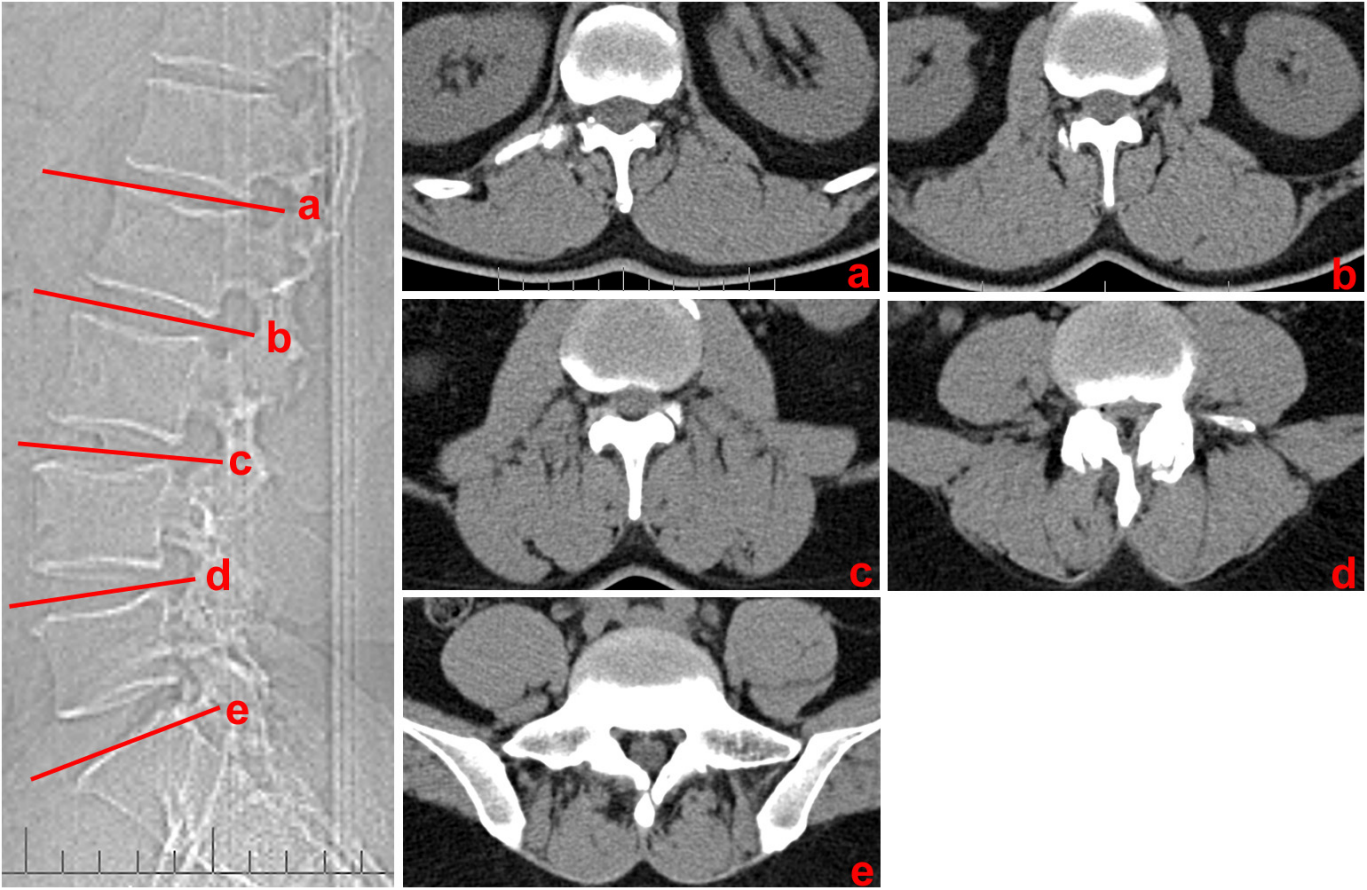
Transverse CT images at different disc level. (a) L1-L2 intervertebral disc level; (b) L2-L3 intervertebral disc level; (c) L3-L4 intervertebral disc level; (d) L4-L5 intervertebral disc level; (d) L5-S1 intervertebral disc level

CT imaging was performed in 30 patients (17 males, 13 females; age range, 18–81 years; mean age, 46 years). All CT images were obtained using a 16-MDCT unit (Siemens Healthcare). The scanning parameters were as follows: 120 kV, 240 mA, 512 × 512 matrix, 3-mm section thickness, 3-mm intersection gap, 40-cm field of view, and soft tissue Kernel. The areas scanned were at the level of the intervertebral disc at L1–L2, L2–L3, L3–L4, L4–L5 and L5–S1, with three sections obtained for each level (Fig 1).

#### Magnetic resonance imaging scans of live patients

**Fig 2:**
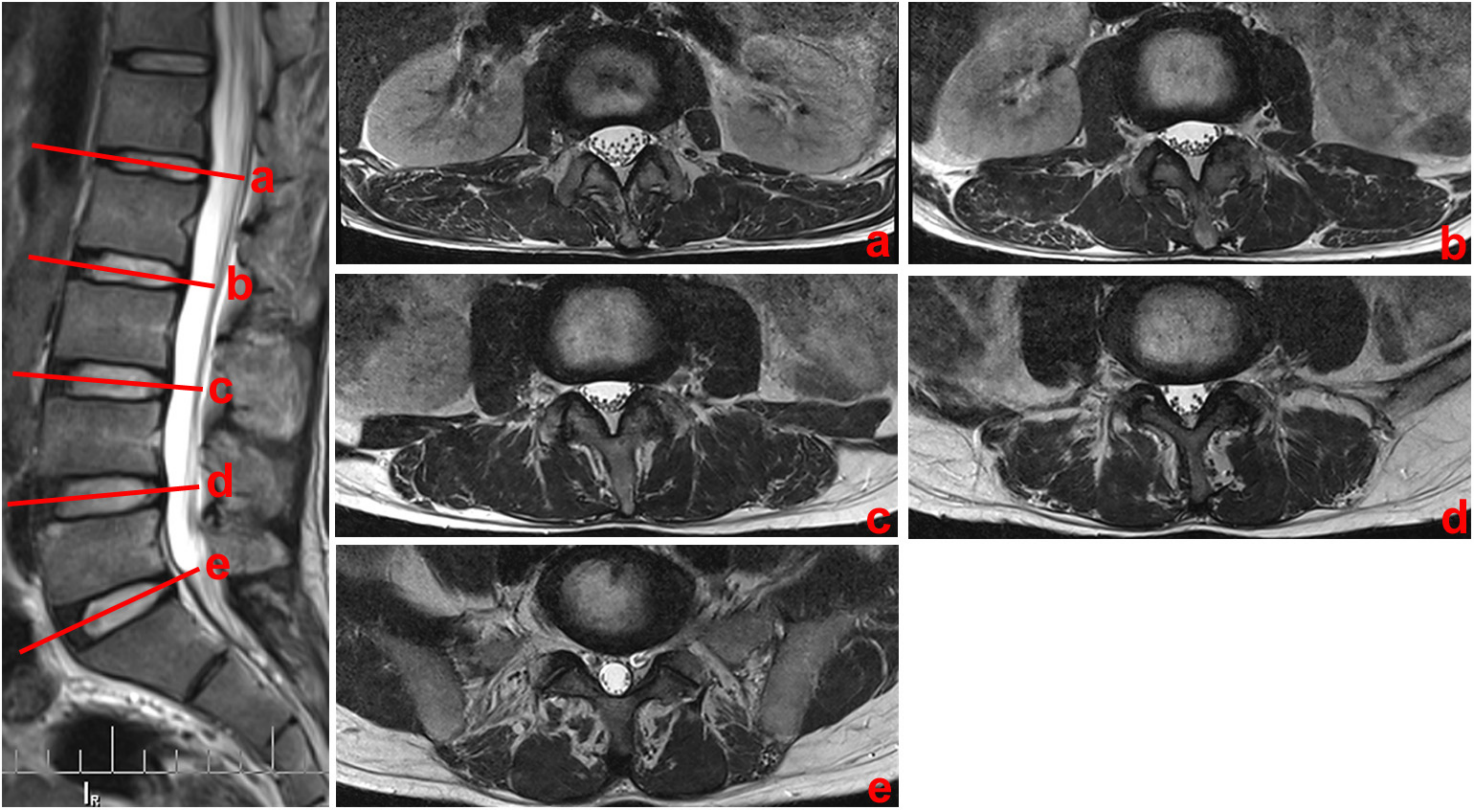
Transverse MR images at different disc level. (a) L1-L2 intervertebral disc level; (b) L2-L3 intervertebral disc level; (c) L3-L4 intervertebral disc level; (d) L4-L5 intervertebral disc level; (d) L5-S1 intervertebral disc level

MRI was performed in 30 patients (16 females, 14 males; age range, 18–74 years; mean age, 49 years). The scanning was based on a 3.0 T MRI unit (Siemens Healthcare) using a 12-element spinal phased array coin. A T2-weighted sequence with a TR/TE of 4000 ms/90 ms was performed to display the loose connective tissue. The scanning parameters were as follows: 256 × 256 matrix, 5-mm section thickness, and 1-mm intersection gap. Scanning was carried out from the L1–L2 to the L5–S1 intervertebral disc levels and with three sections acquired for each level (Fig 2).

### Measurement methods

**Fig 3:**
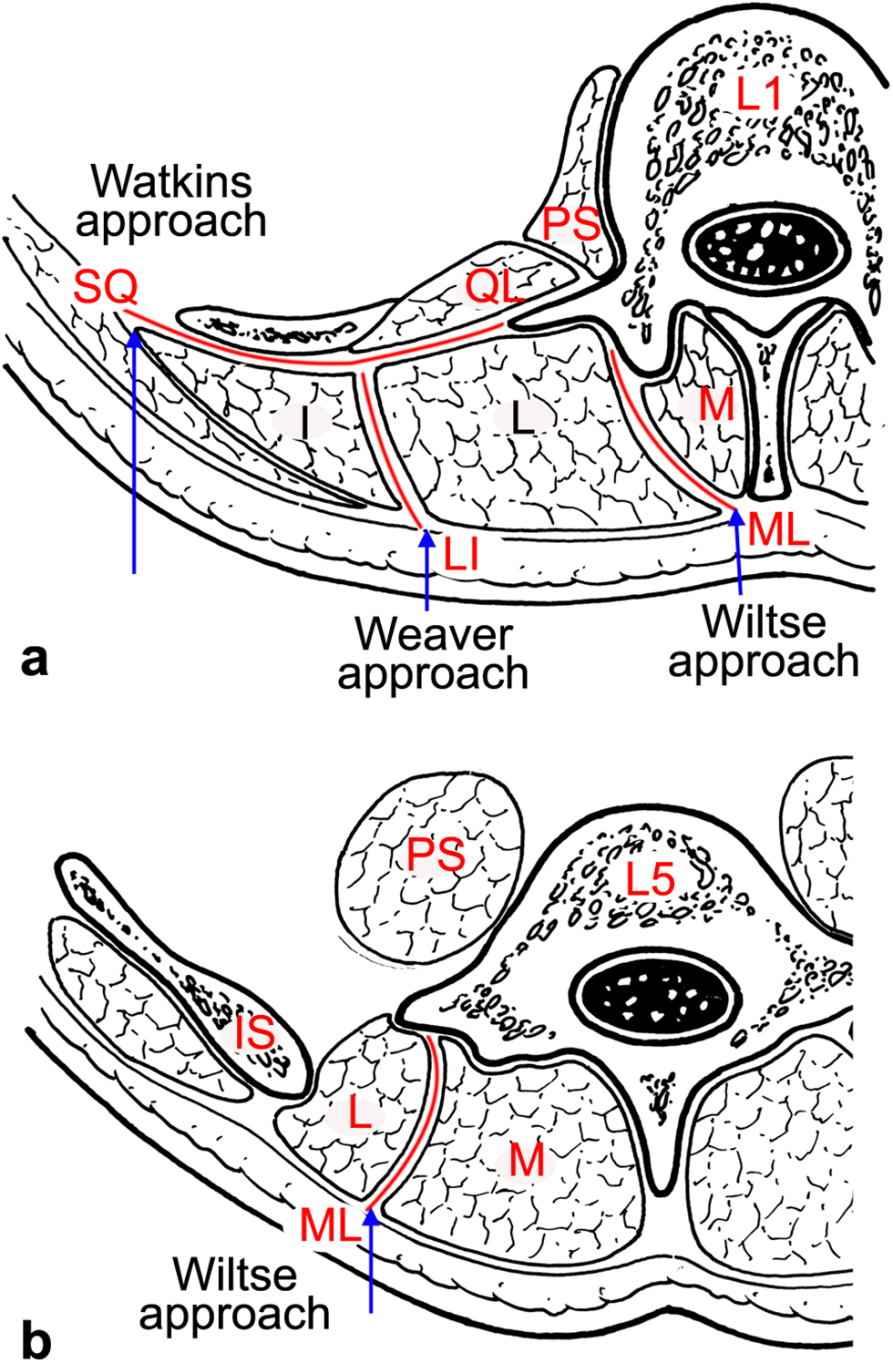
Illustration showing transverse sections at different levels of the lumbar region. (a) At the upper level of the lumbar spine, three cleavage planes within the paraspinal muscles were found: ML, SQ and LI, which are the entries used in the Wiltse, Watkins and Weaver approaches, respectively. (b) At the lower level of the lumbar spine, only ML was found, and its superficial location lied more laterally to the midline than the location at the upper level. The Wiltse approach through ML may be the best choice for protecting muscle integrity and its neurovascular supply. *Arrow*: incision site; *M*: multifidus; *L*: longissimus; *I*: iliocostalis; *IS*: iliac spine; *QL*: quadratus lumborum; *PS*: psoas major.

The measured point corresponded to the incision site at surgery. For the quadratus lumborum (QL) in the Watkins approach, it is easy to come into contact with the boundary of sacrospinalis. As such, the surgical incision was made along the external margin of this muscle and the distance measured was that between the external margin of the sacrospinalis and the midline. For ML in the Wiltse approach and LI in the Weaver approach, the intermuscular space can only be identified by the adjacent muscle. Therefore, the surgical incisions in the middle of the intermuscular spaces were selected for avoiding the injury of the muscle, and the distance measured was that between the midpoint of the incision in the superficial skin and the midline [24]. The guidelines of these incisions are illustrated in Fig 3.

### Standardization

**Fig 4:**
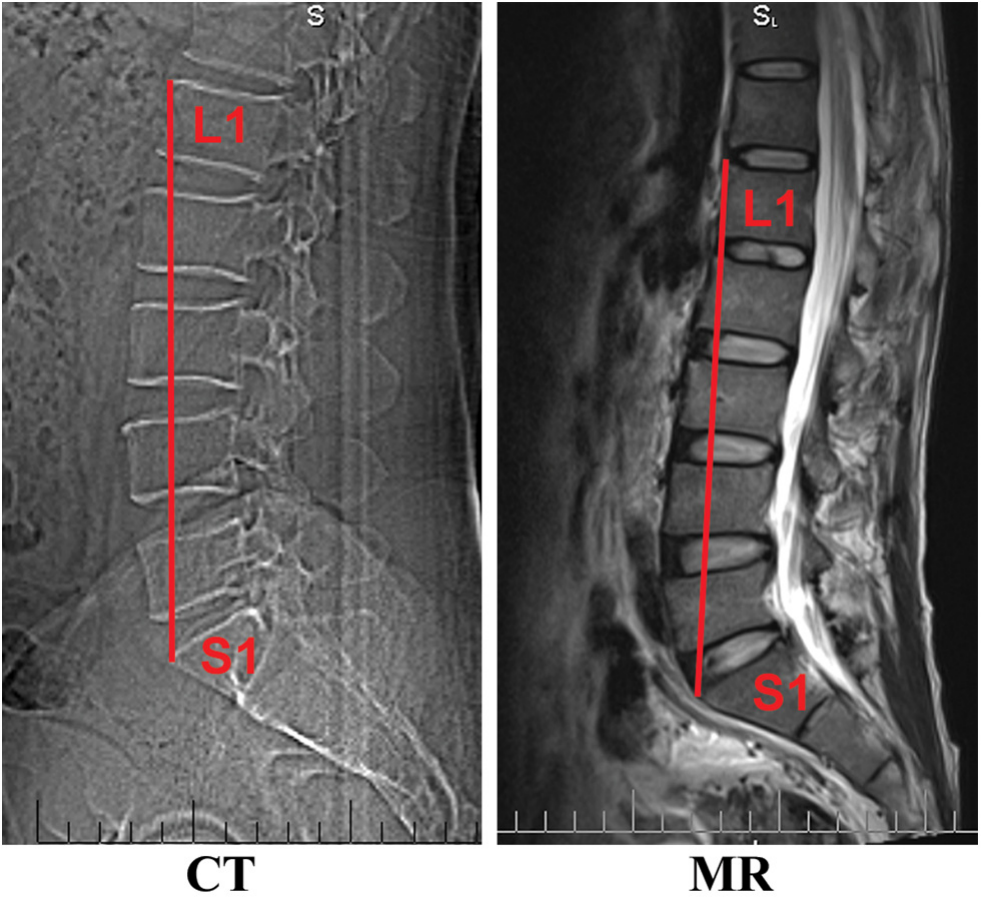
Measurements of the height lumbar vertebrae. The height of lumbar vertebrae was measured by the straight-line distance between the anterior superior border of the L1 vertebra and the anterior superior border of the S1 vertebra (promontory).

The straight-line distance between the anterior superior border of the L1 vertebra and the anterior superior border of the S1 vertebra (promontory) was measured (Fig 4). This distance can reflect differences between individuals of differing heights [25]. The vertebrae was accessed from the abdomen and measured before the sectional anatomy. And then, CT and MRI were performed in post-workstation. The distances based on the anatomy from autopsy, as well as the CT and MRI scan images for all 30 cases in each group were measured, the average values were calculated. Then, these measured distances was multiplied with the height of the individual, and the result was divided by the average value of the group.

### Statistical analysis

Measurements of the intermuscular spaces in the anatomic specimens obtained from the cadavers were made independently by two anatomy specialists and an orthopedic specialist, and followed by computation of their mean values. Each image of the skeletal muscles in patients was double-reviewed and interpreted independently by two musculoskeletal radiologists and a spinal surgeon. Then, the measurements of their intermuscular spaces made. The data used the mean values based on three scan views. Data were analyzed using SPSS19.0 (IBM Corp.) and are presented as the mean ± standard deviation (SD). Statistical comparisons of the distances from the intermuscular cleavage planes to the midline between the various intervertebral disc levels were made using single-factor analysis of variance (ANOVA) followed by the LSD post-hoc test. No difference was found between the right and left sides in both the cadavers and medical images (Paired *t*-test, *P*>0.05). The *P* value < 0.05 was considered to indicate a significant difference.

## Results

### Intermuscular space between multifidus and longissimus varies at different levels of lumbar spine

**Fig 5:**
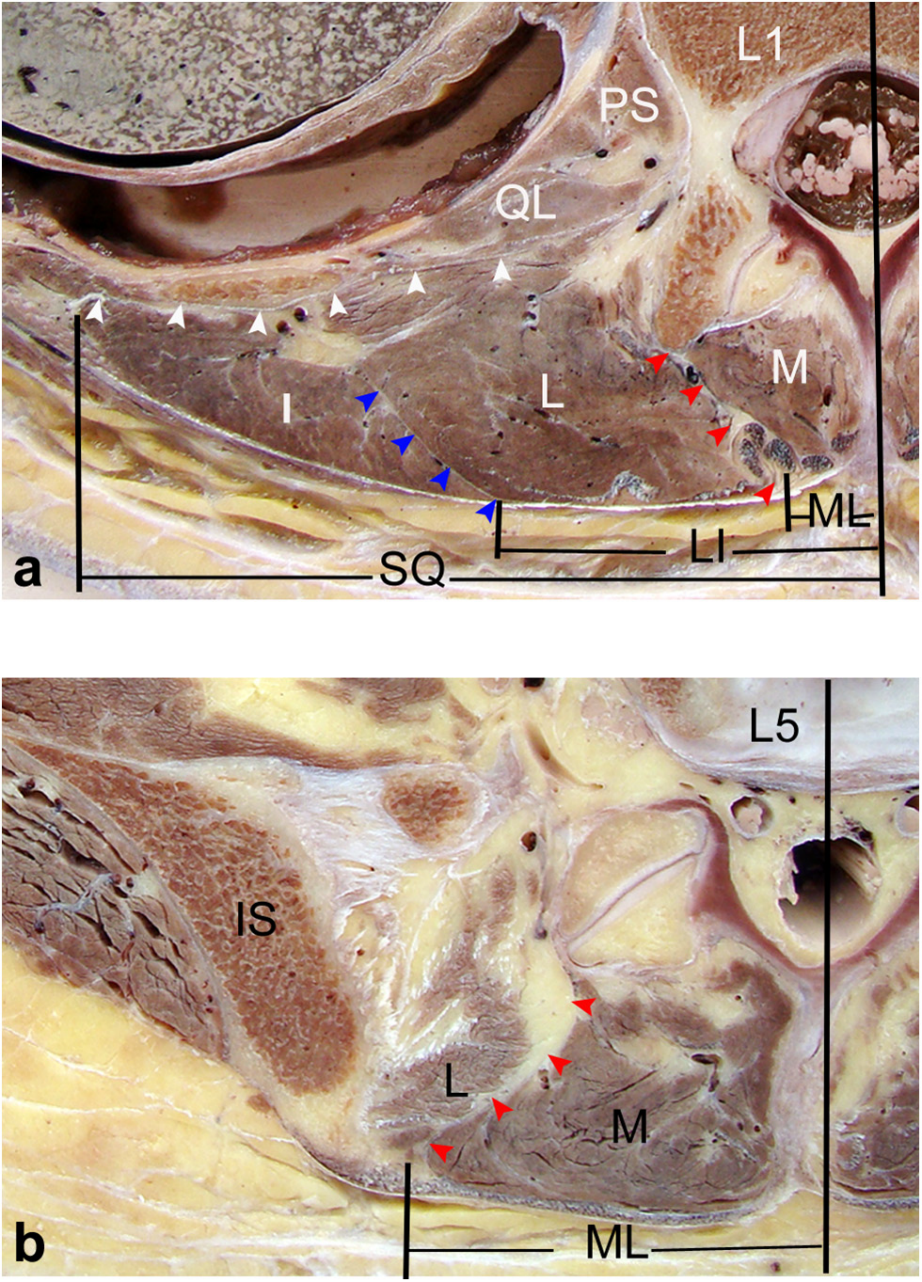
Horizontal views of the posterior lumbar spine in anatomical sections obtained from cadavers. (a) Through the upper level of the lumbar spine (L1), the muscles in the posterior lumbar region were divided into three parts: the medial part (multifidus), middle part (longissimus) and lateral part (iliocostalis). The longissimus and iliocostalis were the two parts of the sacrospinalis. The red arrows point to the ML, which is a natural corridor filled with adipose tissue between the multifidus and longissimus. The blue arrows show the LI, which is a thin fascia between the longissimus and iliocostalis. The white arrows point to SQ between the sacrospinalis and quadratus lumborum. (b) Through the lower level of the lumbar spine (L5), the ML was well defined, and slightly more lateral to the midline. LI and SQ could not be identified. *M*: multifidus; *L*: longissimus; *I*: iliocostalis; *IS*: iliac spine; *QL*: quadratus lumborum; *PS*: psoas major.

In cadavers, the posterior lumbar muscles were divided into three parts: the medial part (multifidus), middle part (longissimus) and lateral part (iliocostalis). At the upper level of the lumbar spine (L1–L3), the intermuscular space between multifidus and longissimus (ML) was clearly identified in all the specimens, and its superficial location is close to the midline with adipose tissue filled in (Fig 5A). At the lower level of the lumbar spine (L4–S1), the ML was also well-defined in all specimens (Fig 5B). From L1-L2 to L5-S1 intervertebral disc level, the mean values between the midline and the ML were 0.8, 1.1, 1.7, 2.9, and 3.5 cm, respectively (Table 1). For each intervertebral disc level, the distance from the intermuscular cleavage plane to the midline was longer than that for the disc level immediately above (*P* < 0.05).

**Table 1:** Comparisons of distances from the intermuscular cleavage planes to the midline between cadavers and patients imaged with CT and MRI.

Intervertebral disc level	Cadavers	Patients, CT	Patients, MRI	F/P
L1–L2				
ML	0.8±0.2 (0.6–1.4)	0.8±0.1 (0.6–1.2)	0.9±0.2 (0.5–1.4)	0.990/0.374
LI	3.4±0.7 (2.2–4.8)	3.3±0.6 (2.2–5.2)	3.4±0.7 (1.9–5.1)	0.240/0.787
SQ	8.1±1.1 (5.9–10.6)	7.9±0.8 (5.5–10.3)	7.9±1.1 (5.7–10.5)	0.396/0.673
L2–L3				
ML	1.1±0.3 (0.5–1.9) ^1^	1.1±0.2 (0.5–1.7) ^1^	1.1±0.3 (0.6–1.8) ^1^	0.184/0.832
LI	3.7±0.8 (2.4–6.0) ^1^	3.6±0.5 (2.5–4.5) ^1^	3.7±0.8 (2.2–5.7) ^1^	0.955/0.387
SQ	7.9±1.1 (6.0–10.2)	7.7±0.9 (5.6–10.1)	7.7±1.1 (5.8–9.9)	0.556/0.575
L3–L4				
ML	1.7±0.5 (0.7–3.3) ^1^ ^2^	1.5±0.5 (0.7–3.1) ^1^ ^2^	1.7±0.5 (0.8–3.1) ^1^ ^2^	1.545/0.216
LI	4.2±0.7 (2.5–5.6) ^1^ ^2^	4.0±0.5 (2.9–5.1) ^1^ ^2^	4.1±0.6 (3.0–5.3) ^1^ ^2^	0.884/0.415
SQ	7.5±1.2 (5.2–9.7) ^1^	7.2±1.0 (4.9–9.1) ^1^ ^2^	7.4±1.0 (5.6–9.2) ^1^	0.885/0.415
L4–L5				
ML	2.9±0.5 (1.9–4.4) ^1^ ^2^ ^3^	2.9±0.6 (1.4–4.3) ^1^ ^2^ ^3^	2.8±0.6 (1.6–4.2) ^1^ ^2^ ^3^	0.811/0.446
L5–S1				
ML	3.5±0.6 (2.2–4.8) ^1^ ^2^ ^3^ ^4^	3.4±0.5 (2.5–4.6) ^1^ ^2^ ^3^ ^4^	3.4±0.7 (1.9–4.6) ^1^ ^2^ ^3^ ^4^	1.040/0.356

Data are presented as the mean ± SD (range).

^1^ Compared with L1-L2 level, *P*<0.05.

^2^ Compared with L2-L3 level, *P*<0.05.

^3^ Compared with L3-L4 level, *P*<0.05.

^4^ Compared with L4-L5 level, *P*<0.05.

**Fig 6:**
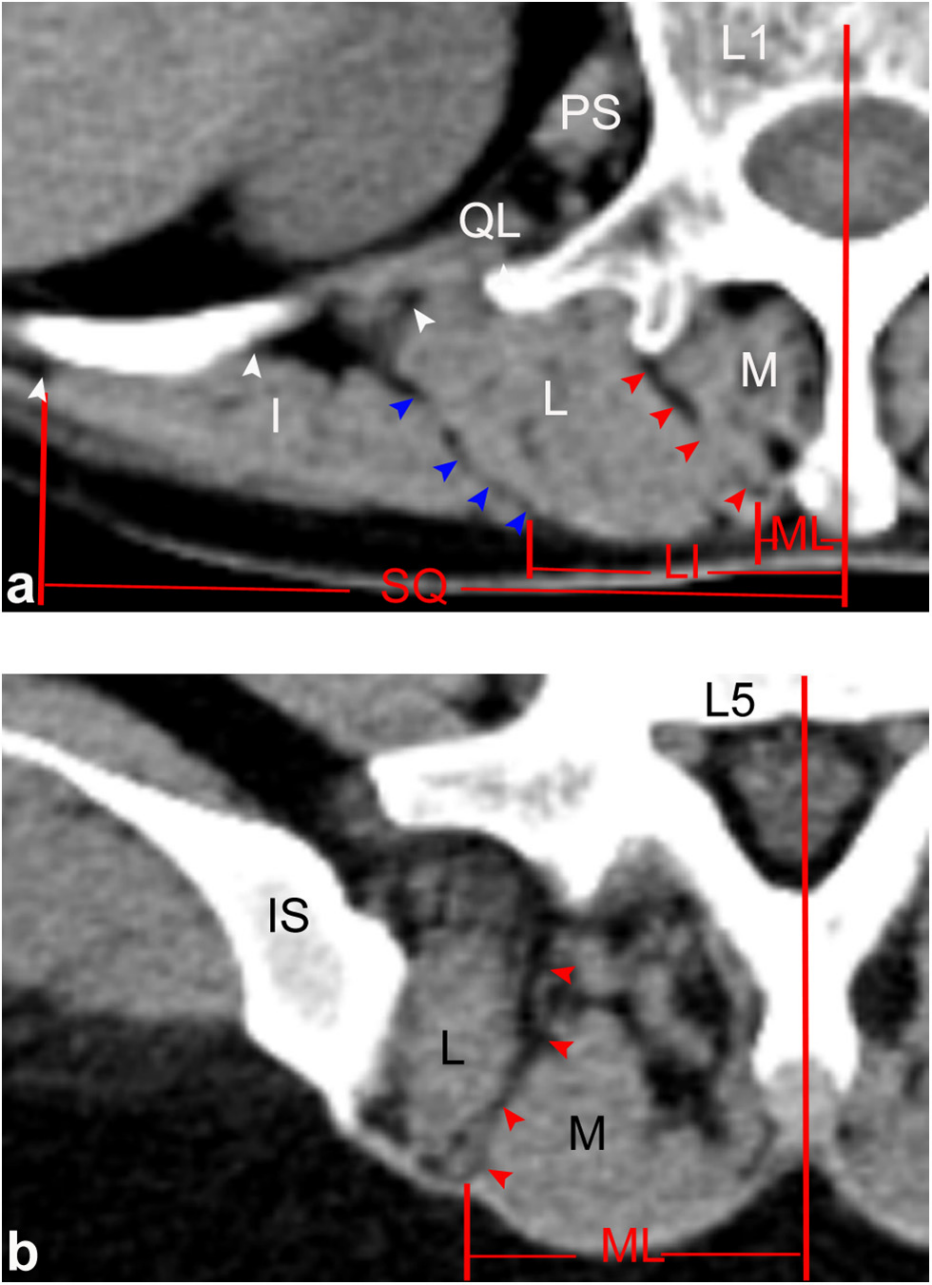
Horizontal views of the posterior lumbar spine in CT images. (a) Through the upper level of the lumbar spine (L1), ML (red arrows), SQ (white arrows) and LI (blue arrows) were identified by line-shaped low densities within the paraspinal muscles. (b) Through the lower level of the lumbar spine (L5), only the ML was identified. *M*: multifidus; *L*: longissimus; *I*: iliocostalis; *IS*: iliac spine; *QL*: quadratus lumborum; *PS*: psoas major.

**Fig 7:**
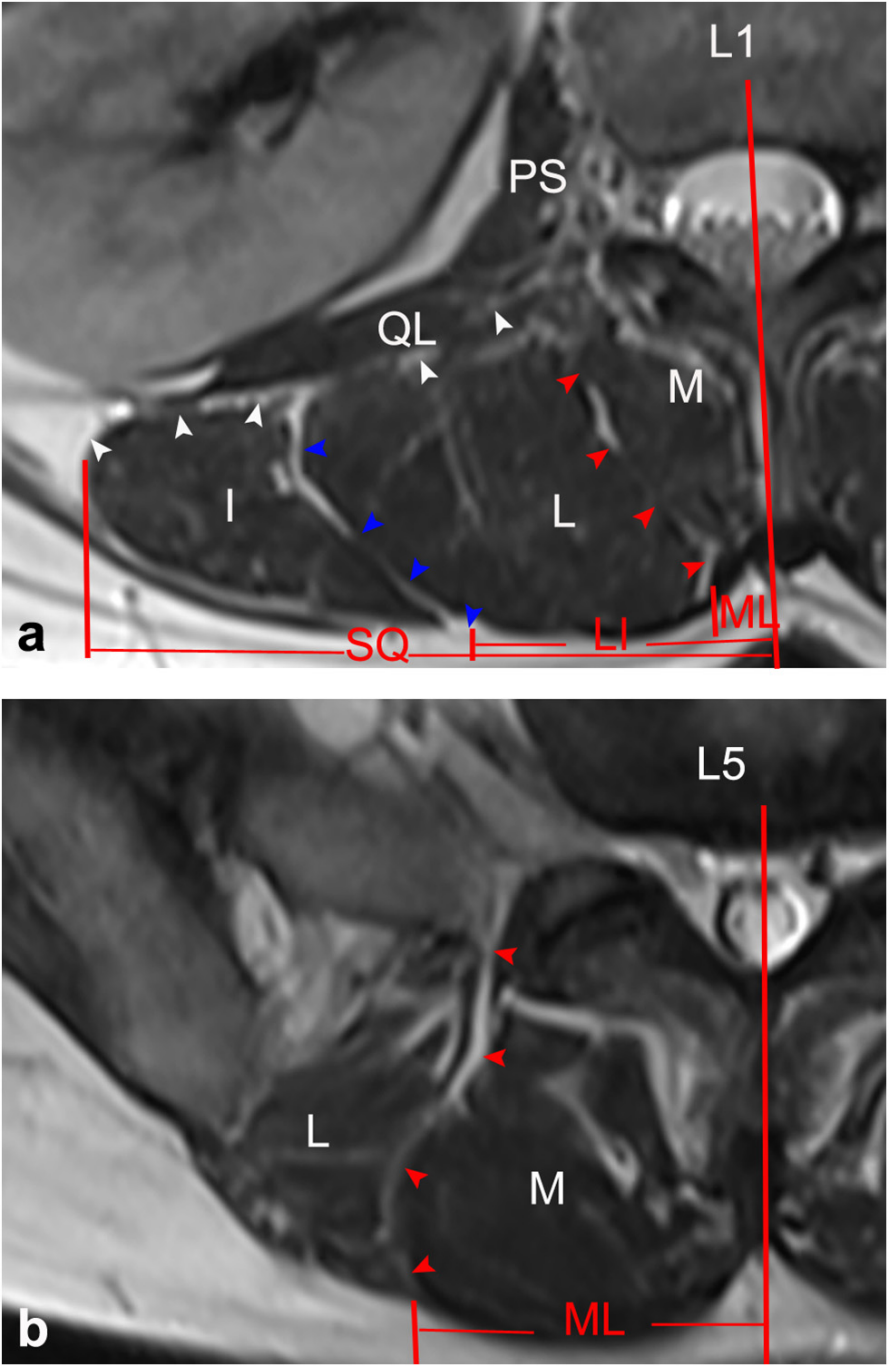
Horizontal views of the posterior lumbar spine in MRI images. (a) Through the upper level of the lumbar spine (L1), ML (red arrows), SQ (white arrows) and LI (blue arrows) were identified by lines of high signal intensity inserted into the paraspinal muscles. (b) Through the lower level of the lumbar spine (L5), only the ML was identified. *M*: multifidus; *L*: longissimus; *I*: iliocostalis; *IS*: iliac spine; *QL*: quadratus lumborum; *PS*: psoas major.

In patients, the ML also varied at different levels of the lumbar spine and showed a low discontinuous density in CT (Fig 6) as well as a high signal in MRI (Fig 7). Based on the CT images, from the L1-L2 to L5-S1 intervertebral disc level, the mean values between the midline and the ML were 0.8, 1.1, 1.5, 2.9, and 3.4 cm, respectively. In MRI images, the corresponding mean values were 0.9, 1.1, 1.7, 2.8, and 3.4 cm, respectively. There were not any statistical difference found between cadavers, CT and MRI images (Table 1).

### Intermuscular space between sacrospinalis and quadratus lumborum do not exist in all of the lumbar spine

In cadavers, the intermuscular space between sacrospinalis and quadratus lumborum **(**SQ) between the lateral border of the sacrospinalis and the quadratus lumborum was formed by a sheet of aponeurosis. At the upper level of the lumbar spine (L1–L3), the SQ was clearly identified in all the specimens, and it courses posterolaterally to merge with the lumbar fascia and medially to attach the transverse process (Fig 5A). At the lower level of the lumbar spine (L4–S1), the sacrospinalis was confined by the multifidus medially and posterior superior iliac spine laterally, and the SQ did not exist at the lower level of the lumbar spine (Fig 5B). From L1-L2 to L3-S4 intervertebral disc level, the mean values between the midline and the SQ were 8.1, 7.9, and 7.5 cm, respectively (Table 1).

In patients, the SQ was only identified at the upper level of the lumbar spine as well, which showed a low discontinuous density in CT (Fig 6) and a high signal in MRI (Fig 7). From the CT images, from L1-L2 to L3-S4 intervertebral disc level, the mean values between the midline and the SQ were 7.9, 7.7, and 7.2 cm, respectively. For the MRI images, the corresponding mean values were 7.9, 7.7, and 7.4 cm, respectively. There were no statistical difference found between the data extracted from autopsy, CT, and MRI scanning (Table 1).

### Intermuscular space between longissimus and iliocostalis exists at the upper level of the lumbar spine

Based on the cadavers, the intermuscular space between longissimus and iliocostalis **(**LI) was interposed with a thin and membranous fascia. Posteriorly, the fascia was continuous with the posterior layer of the lumbar fascia and its trajectory parallels ML. Anteriorly, it was fused to the middle layer of the lumbar fascia (Fig 5A). At the upper level of the lumbar spine (L1–L3), the LI was clearly identified in all the specimens. At the lower level of the lumbar spine (L4–S1), the tendons of the longissimus and iliocostalis muscles merged into the aponeurosis which is inserted into the superomedial iliac crest, and the LI was not identified in any specimens (Fig 5B). From L1-L2 to L3-S4 intervertebral disc level, the mean values between the midline and the LI were 3.4, 3.7 and 4.2 cm, respectively (Table 1). For each intervertebral disc level, the distance from the intermuscular cleavage plane to the midline was longer than that for the disc level immediately above (*P*<0.05).

From our patients, the LI was also only identified as a discontinuous density in CT (Fig 6) or a high signal in MRI (Fig 7) at the upper level of the lumbar spine. Using the CT images, from L1-L2 to L3-S4 intervertebral disc level, the mean values between the midline and the SQ were 3.3, 3.6, and 4.0 cm, respectively. From the MRI images, the corresponding mean values were 3.4, 3.7, and 4.1 cm, respectively. There were no statistical difference found between the data from our cadavers, CT, and MRI scans (Table 1).

## Discussion and Conclusion

To the best of our knowledge, the present study is the first to provide a focused description of the surgical anatomy of the posterior lumbar muscles with respect to natural cleavage planes at different intervertebral disc levels. These cleavage planes contain three intermuscular spaces, ML, SQ and LI, which are the entries used in the Wiltse approach, the Watkins approach, and the Weaver approach, respectively. The configuration of the intermuscular spaces in the lumbar spine was shown clearly in the thin transverse sections obtained from cadavers and these structures were well matched with CT and MRI images obtained in patients. In both patients and cadavers, the configuration of the intermuscular planes differed between the upper and lower levels of the lumbar spine, with ML, LI and SQ all discernable at disc levels L1–L2, L2–L3 and L3–L4, but only ML present at L4–L5 and L5–S1. Our medical imaging procedures have clearly confirmed these findings. Therefore, CT and MRI are suitable medical imaging modalities for investigating and determining the location of the natural cleavage planes of the paraspinal muscles in patients. As such, it can provide useful guidance to surgeons carrying out minimally-invasive surgical procedures on the lumbar spine.

### Is the paramedian incision the only choice in the Wiltse approach?

The multifidus is believed to be the major posterior stabilizing muscle of the spine [26, 27]. To minimize trauma to this muscle, Wiltse introduced a muscle-splitting approach between multifidus and longissimus in 1968, with two vertical incisions made bilaterally 30 mm from the midline [17]. This approach has received support from several other specialists who have described the detailed anatomy around this intermuscular space [28, 29]. Due to esthetic arguments and potential difficulties in case of iterative surgery, in 1988, Wiltse recommended the use of a single median incision [18]. The present study demonstrates that in both cadavers and patients, the superficial location lies close to the midline at the upper lumbar region. At the L1–L2 and L2–L3 intervertebral disc levels, the mean distances between the midline and ML were 0.8 cm and 1.1 cm and no more than 1.7 cm. On the basis of our results, a single midline incision should be made in situations that warrant bilateral exposure. However, at the L4–L5 and L5–S1 levels, the ML lays substantially further away from the midline with the mean distance between the midline and ML at about 3 cm. To minimize muscle dissection and preserve neurovascular and tendon integrity, it is easier to use an equidistant paramedian incision and create separate working corridors for the performance of bilateral procedures.

### Is the Watkins approach ideal for whole lumbar spine surgery?

In 1953, the Watkins approach between the lateral border of sacrospinalis and quadratus lumborum was described as a novel technique for spinal fusion [15]. More recently, it has also been used for removing far lateral disc herniation [30]. The reported advantage of the procedure is that it provides more adequate exposure of the posterolateral vertebral elements. However, it also involves bone removal and detachment of muscle that do not conform to the standard of minimally-invasive surgery [31]. Our results indicate that the SQ is interposed with a thin fascia at the upper levels of the lumbar spine (L1–L3), allows direct access to the muscular cleavage plane without subcutaneous detachment, and is compatible with the concept of inducing minimal trauma. However, at the lower levels of the lumbar spine (L4–S1), the SQ does not exist. To gain satisfactory access to this region, the iliac attachment of the muscles of the spine should be released with an osteotome and a thin layer of the ilium can be removed. Exposure can then be enlarged by blunt dissection to protect the iliohypogastric nerves. All these surgical procedures can lead to disruption of muscle integrity or damage to its neurovascular supply. Consequently, we suggest that the ML is superior to SQ for lateral access to the facet and transverse process at the lower lumbar spine in order to protect muscle integrity and neurovascular supply.

### Is the intermuscular space between longissimus and iliocostalis a safe natural cleavage plane for lumbar surgery?

It is generally believed that there are only two intermuscular spaces (ML and SQ) that are available for the paraspinal approach. Recently, Weaver described an intermediately lateral approach directed obliquely to the area of the pedicle via a natural plane existing within the longissimus and iliocostalis muscle complex [19]. This was referred to as the Weaver approach in Cheng’s study [32]. The authors concluded that this plane provides a much less invasive access to the lateral spine from L3 to S2. In contrast to the Wiltse’s approach, this intermuscular space creates a working corridor that fully preserves muscle fibers and the tendinous attachments of multifidus. Our results also suggest that at the upper lumbar region, a thin layer of fascia exists between longissimus and iliocostalis. However, contrary to the view of Weaver, this natural intermuscular space does not exist in the lower lumbar region based on the fact that the tendons of longissimus and iliocostalis merge into the aponeurosis which is inserted into the superomedial iliac crest. Therefore, LI provides ideal access to the mid-lumbar region (L2–4), but not to the lower lumbar spine. In addition, the mean distances between the midline and LI at different levels were approximately 4 cm. To reduce the incision degree of elasticity, the paramedian skin incision should be made at the level of the upper lumbar spine without cutting the nerve to longissimus [33].

### Role of computed tomography and magnetic resonance imaging in preoperative clinical decision-making

The intermuscular space is filled with loose connective tissue and can be reliably identified by preoperative CT or MRI. The cleavage planes showed a low discontinuous density within neighboring muscles in CT scans and a high signal in MRI. Knowledge of the superficial locations of the various intermuscular spaces allows for skin incisions to be made with a higher degree of precision. Our study determined all the distances from the intermuscular spaces to the midline at different intervertebral disc levels. No significant differences were statistically found between measurements in cadavers and those made using CT and MRI scans of patients. However, the individual values varied in different patients, highlighting the need to establish the actual value in each patient undergoing surgery. Therefore, preoperative imaging of patients will facilitate selection of the muscle-splitting approach to the lumbar spine. This can then allow for the incision to be made with a higher degree of precision.

### Limitations

The present study has its limitations. Firstly, the sample size was relatively small and thus additional studies are merited to confirm the results. Secondly, CT and MRI scanning of the cadavers were not performed, which precludes direct comparison of imaging data between cadavers and patients. It seemed more direct and accurate to obtain the data we need via autopsy instead of using the medical imaging of cadavers.

## Supporting Information

S1 File The relevant data about this manuscript. (XLS)
